# WNT5A Interacts With FZD5 and LRP5 to Regulate Proliferation and Self-Renewal of Endometrial Mesenchymal Stem-Like Cells

**DOI:** 10.3389/fcell.2022.837827

**Published:** 2022-02-17

**Authors:** Tianqi Li, Rachel W.S. Chan, Cheuk-Lun Lee, Philip C.N. Chiu, Raymond H.W. Li, Ernest H.Y. Ng, William S.B. Yeung

**Affiliations:** ^1^ Department of Obstetrics and Gynaecology, LKS Faculty of Medicine, The University of Hong Kong, Hong Kong, Hong Kong SAR, China; ^2^ Shenzhen Key Laboratory of Fertility Regulation, The University of Hong Kong Shenzhen Hospital, Shenzhen, China

**Keywords:** endometrium, adult stem cells, wnt / b-catenin, wnt5a, stem cell niche

## Abstract

Endometrial mesenchymal stem-like cells (eMSC) reside in the basal layer of the endometrium and are responsible for cyclic regeneration during the reproductive lives of women. Myometrial cells act as a component of the niche and regulate the stem cell fate through the activation of WNT/β-catenin signaling *via* WNT5A. Since WNT5A-responsive mechanisms on eMSC are still uncertain, we hypothesize that the WNT ligand–WNT5A works to activate WNT/β-catenin signaling through binding to Frizzled receptors (FZDs) and co-receptor low-density lipoprotein receptor-related protein 5 (LRP5). Among the various receptors that have been reported to interact with WNT5A, we found FZD5 abundantly expressed by eMSC when compared to unfractionated stromal cells. Neutralizing the protein expression by using anti-FZD5 antibody suppressed the stimulatory effects on phenotypic expression and the clonogenicity of eMSC in a myometrial cell–eMSC co-culture system as well as in an L-Wnt5a conditioned medium. Gene silencing of FZD5 not only reduced the binding of WNT5A to eMSC but also decreased the TCF/LEF transcriptional activities and expression of active β-catenin. Inhibition of LRP coreceptors with recombinant Dickkopf-1 protein significantly reduced the binding affinity of eMSC to WNT5A as well as the proliferation and self-renewal activity. During postpartum remodeling in mouse endometrium, active β-catenin (ABC) was detected in label-retaining stromal cells (LRSCs), and these ABC^+^ LRSCs express FZD5 and LRP5, suggesting the activation of WNT/β-catenin signaling. In conclusion, our findings demonstrate the interaction of WNT5A, FZD5, and LRP5 in regulating the proliferation and self-renewal of eMSC through WNT/β-catenin signaling.

## Introduction

Human endometrium undergoes cyclical changes between proliferation, differentiation, and shedding under the control of sex steroids (Jabbour et al., 2006; Gellersen and Brosens, 2014). Endometrial stem/progenitor cells are responsible for the cyclic regenerative capacity of the human endometrium (Chan et al., 2004; Gargett et al., 2016). A specific population of endometrial stromal cells termed endometrial mesenchymal stem cells (eMSC) localized in the perivascular regions of functionalis and basalis can be identified by the coexpression of CD140b and CD146 (Schuring et al., 2011). These cells are clonogenic, express typical BM-MSC phenotypic markers (CD29, CD44, CD73, CD90, and CD105), and are able to differentiate into the mesoderm lineages upon specific induction media (Schwab and Gargett, 2007; Spitzer et al., 2012). The percentage of eMSC was similar across the menstrual cycle, but interestingly, eMSC from menstrual underwent more rounds of self-renewal and enabled a greater total cell output than those from the secretory phase (Xu et al., 2017).

The WNT signaling pathway is a complicated and highly conserved signaling cascade that can be categorized into a β-catenin-dependent (WNT/β-catenin signaling) and β-catenin-independent signaling pathway (Wend et al., 2010). WNT/β-catenin signaling is a well-elucidated pathway that is featured in various self-renewing tissues and is involved in somatic stem cell maintenance (Nusse and Clevers, 2017; Steinhart and Angers, 2018). WNT/β-catenin signaling is activated by the interaction of WNT ligand with a functional receptor from the Frizzled family (FZD) and a coreceptor low-density lipoprotein receptor-related protein (LRP) (Nusse, 2005; Gordon and Nusse, 2006; Dijksterhuis et al., 2015). The WNT-FZD-LRP trimeric complex results in the inhibition of β-catenin phosphorylation and leads to the stabilization of β-catenin, which is then translocated to the nucleus and binds to transcription factors T-cell factor (TCF) and lymphoid enhancer-binding factor (LEF) for stimulating the transcription of WNT target genes (Cadigan and Waterman, 2012). Meanwhile, β-catenin-independent signaling can be further subcategorized into the planar cell polarity pathway or the WNT/Ca^2+^ pathway according to its downstream effectors (Komiya and Habas, 2008).

Somatic stem cells reside in the niche. The neighboring niche cells provide soluble, adhesive, and physical signals to the stem cells, which are crucial for maintaining stem cell functions (Chacón-Martínez et al., 2018). Our group has demonstrated that WNT5A derived from myometrial cells can regulate eMSC by activating the WNT/β-catenin signaling pathway (Cao et al., 2019). To gain a better understanding of the WNT5A-responsive manner, we propose that specific FZD receptor(s) and LRP coreceptor(s) expressed on eMSC will bind with WNT5A to activate the WNT/β-catenin signaling pathway. Here, we report the expression of WNT5A-related FZD receptors and WNT coreceptors in different subpopulations of endometrial stromal cells and demonstrate the role of FZD5 and LRP5 in the WNT5A-induced activation of WNT/β-catenin signaling in eMSC.

## Materials and Methods

### Human Tissues

Full-thickness human endometrial tissue and underlying myometrium were collected from 41 women aged between 38 and 53 years (mean age 46 years) who had regular menstrual cycles and underwent total abdominal hysterectomy for benign pathologies (Supplementary Table S1). The recruited subject took no exogenous hormonal therapy for at least 3 months prior to surgery. Written consents were obtained from all women before their participation of the research. Ethical approval was obtained from the Institutional Review Board of The University of Hong Kong/Hospital Authority Hong Kong West Cluster UW14-133 and UW19-851.

An experienced histopathologist defined the phase of the menstrual cycle as proliferative (n = 23) and secretory (n = 18) based on microscopic morphology in hematoxylin and eosin-stained tissue sections.

### Isolation of Endometrial Cells

Endometrial tissue was minced and digested with phosphate-buffered saline (PBS) containing 0.3 mg/ml collagenase III (Worthington Biochemical Corporation, Freehold, NJ, United States) and 40 μg/ml deoxyribonuclease type I (Worthington Biochemical Corporation) in a shaking water bath for 60 min at 37°C (Xiang et al., 2014). The endometrial tissue underwent two rounds of digestion, and the dispersed cells were filtered through 40-μm sieves (BD Bioscience, San Jose, CA, United States). The red blood cells, cell debris, and cell clumps were removed by Ficoll-Paque (GE Healthcare, Uppsala, Sweden) density-gradient centrifugation. Anti-CD45 antibody-coated Dynabeads (Invitrogen, Waltham, MA, United States) were used for the removal of leukocytes, and anti-CD326 antibody-coated microbeads (Miltenyi Biotec Inc., San Diego, CA, United States) were used to separate epithelial cells from stromal cell population. Freshly purified stromal cells were seeded onto 10-cm dishes (BD Biosciences, San Jose, CA, United States) coated with fibronectin (1 mg/ml, Invitrogen) containing growth medium (GM) with 1% penicillin (Invitrogen), 1% L-glutamine (Invitrogen), and 10% FBS (Invitrogen) in DMEM/F-12 (Sigma-Aldrich, St Louis, MA, United States). Stromal cells were cultured for 1–2 weeks in a humidified carbon dioxide incubator at 37°C. The medium was changed every 7 days.

### Magnetic Selection of Endometrial Mesenchymal Stem-Like Cells

To obtain eMSC (CD140b^+^CD146^+^ cells), two separate positive magnetic bead selections were conducted (Xu et al., 2017). After *in vitro* expansion, stromal cells were trypsinized and incubated with PE-conjugated anti-CD140b antibody (R&D Systems, Minneapolis, MN, United States) for 45 min at 4°C and then with antimouse IgG1 coated magnetic microbeads (Miltenyi Biotec Inc.) for 15 min at 4°C. The CD140b^+^ population was obtained by applying the cell suspensions to MS columns (Miltenyi Biotec Inc.) in a magnetic field. The CD140b^+^ stromal cells were cultured in fibronectin-coated plates containing GM at 37°C in 5% CO_2_ for 7–10 days to allow microbead degradation during cell expansion. When the plates were 80% confluent, the cells were trypsinized and incubated with anti-CD146 antibody-coated microbeads (Miltenyi Biotec Inc.) for 15 min at 4°C. CD140b^+^CD146^+^ eMSC were obtained from the column separation. Phenotypic study of eMSC shows their positive expression for CD140b and CD146 (Cao et al., 2021). Stromal cells at passages 1–3 were used in this study.

### Isolation of Myometrial Cells

Myometrial tissue was minced into 1-mm^3^ pieces and dissociated in PBS containing collagenase type III (300 μg/ml) and deoxyribonuclease type I (40 μg/ml) in a shaking water bath for 4 h at 37°C as described (Cao et al., 2019). Single cells were filtered through 100-µm sieves (BD Bioscience) and seeded into 100-mm dishes containing GM. Upon ∼80% confluence, the myometrial cells were trypsinized and passaged. Myometrial cells at passages 1–3 were used in this study.

### Coculture

eMSC were seeded at clonal density (300 cells per well) into a 6-well plate, and myometrial cells pretreated with mitomycin C (2.7 × 10^4^) were seeded into transwell insert (EMD Millipore, Billerica, MA, United States) and cultured separately. On the following day, the inserts were placed into the 6-well plates with eMSC for indirect coculture. All treatment groups were set up in duplicates, and eMSC cultured alone (Monoculture) served as the control. The culture medium was changed every 7 days.

### *In Vitro* Colony-Forming Assay

After 15 days of culture, eMSC were fixed in 10% formalin, washed, and stained with Toluidine Blue (1 mg/ml, Sigma-Aldrich). Clonogenic efficiency was calculated as the number of colonies formed divided by the number of cells seeded multiplied by 100 (Cao et al., 2019).

### Preparation of Myometrial Conditioned Medium

For myometrial conditioned medium, 1 × 10^6^ cells were cultured in T-75 flasks for 3 days in GM, washed with PBS, and then cultured in 5 ml of serum-free medium (DMEM-F12 base medium). The myometrial conditioned medium (CM) was collected after 24 h and was filtered through a 0.45-μm filter. The CM is concentrated using an Amicon^®^ Ultra-15 centrifugal filter (EMD Millipore) with a molecular weight cutoff of 10 kDa and centrifugation at 4,000 *g* for 45 min at 4°C. The myometrial concentrated conditioned medium (Myometrial CCM) was stored at −80°C. The amount of proteins from 5 ml of myometrial CM was considered as one unit of myometrial CCM proteins. Myometrial CCM at 1/6 unit was used for various bioassays (Cao et al., 2019). CCM collected from cells free DMEM-F12 medium was used as control.

### Immunofluorescence Staining, RNA Extraction, Reverse Transcription, Quantitative Polymerase Chain Reaction, Western Blotting, and Flow Cytometry

The protocols are described in Supplementary Material Methods and Supplementary Table S2–S4.

### Activation of WNT Signaling

L-Wnt5a CM was obtained from spent cultured medium of mouse fibroblasts L-M (TK-) cells transfected with a Wnt5a vector (CRL-2814, ATCC, Atlanta, United States). Mouse L-Wnt5A cells (∼2 × 10^6^ cells) were cultured in a T-75 flask containing GM until they reached 80% confluence. The following day, serum-free medium was replaced, cultured for 24 h and collected after sterile filtration as L-Wnt5A CM. Control CM was obtained from normal mouse fibroblast L-M (TK-) cells. All CMs were stored at −80°C. The ratio of 1:10 diluted WNT5A CM was used. Recombinant human WNT5A (0.01 μg/ml, R&D Systems) was supplemented to the GM of eMSC.

### Gene Silencing and Luciferase Assay

eMSC were seeded in 48-well plates at a density of 1 × 10^4^ cells/well in OptiMEM (Invitrogen). The next day, the cells were transfected with 10 pmol of siRNA directed against *FZD5* (s15416 and s15417, Ambion) or siRNA control with scrambled sequence (Ambion) using 10 pmol of a Lipofectamine RNAiMax transfection reagent (Invitrogen). On the following day, the cells were cotransfected with 4 μg of a TOP-flash/FOP-flash vector and 1 μg of pRL-TK (Renilla- TK- luciferase vector, Promega, Madison, WI, United States) using Lipofectamine 3,000 (Invitrogen). Eighteen hours after transfection, the medium was changed to growth medium, and some wells were treated with L-cell or L-Wnt5A CM or myometrial CCM or human recombinant WNT5A (0.01 μg/ml, R&D Systems) or human recombinant dickkopf-1 (0.2 μg/ml, DKK-1, R&D systems) for 48 h. The luciferase activity was measured using a GLOMAX™ 96 microplate luminometer. The TOP/FOP ratio was calculated. The knockdown efficiency was assessed by flow cytometry (Supplementary Figure S1A).

### Protein Labeling Assay

Human recombinant WNT5A protein (20 μg, 645-WN/CF, R&D systems) was labeled with a fluorescence dye using the Alexa Fluor 488 microscale protein labeling kit according to the manufacturer’s instruction (Thermo Fisher Scientific). eMSC were transfected with 10 pmol of siRNA directed against *FZD5* or scrambled siRNA as described above. The next day, cells were harvested and stained with Alexa Fluor 488 labeled human recombinant WNT5A protein for 2 h at 4°C in the dark. Another set of cells were treated with recombinant DKK-1 (0.2 μg/ml, R&D systems) for 2 days. Cells were washed and analyzed by a CytoFlex™ flow cytometer (Beckman Coulter, Ca, United States). Data were analyzed with the FlowJo software (Tree Star Inc., OR, United States).

### Label-Retaining Cells in Mouse Endometrium

Mouse endometrial paraffin sections were obtained from our previous label-retaining cell (LRC) experimental setup (Cao et al., 2015). In brief, day 19 prepubertal CD57BL/6J female mice were administered with bromodeoxyuridine (BrdU) intraperitoneally (50 μg/g of body weight; Sigma Aldrich) twice daily for four consecutive days and allowed to grow without further labeling. After sexual maturity age, some of the mice were sacrificed at 6 weeks chased (n = 4). Other animals were mated with > 6-week-old fertile C57BL/6J male mice, and the presence of the vaginal plug indicates that the female mice are pregnant. These mice were sacrificed ∼8 weeks chase (postpartum day 1). All animals were euthanized, uterine horns collected, fixed overnight with 4% paraformaldehyde, and processed into paraffin blocks by standard method.

### Statistical Analysis

Data were analyzed using the GraphPad PRISM software (version 8.0; GraphPad Software Inc., San Diego, CA, United States) and tested for normal distribution using D’Agostino and Pearson test. Mann–Whitney test was performed to determine the statistical significance between two groups. Kruskal–Wallis test followed by Dunn’s *post hoc* test was used for multiple group comparisons. Data are presented as mean ± standard deviation (SD). *p* < 0.05 was considered statistically significant.

## Results

### Expression of WNT-Related Receptors in Different Subpopulations of Human Endometrial Stromal Cells

**FIGURE 1:**
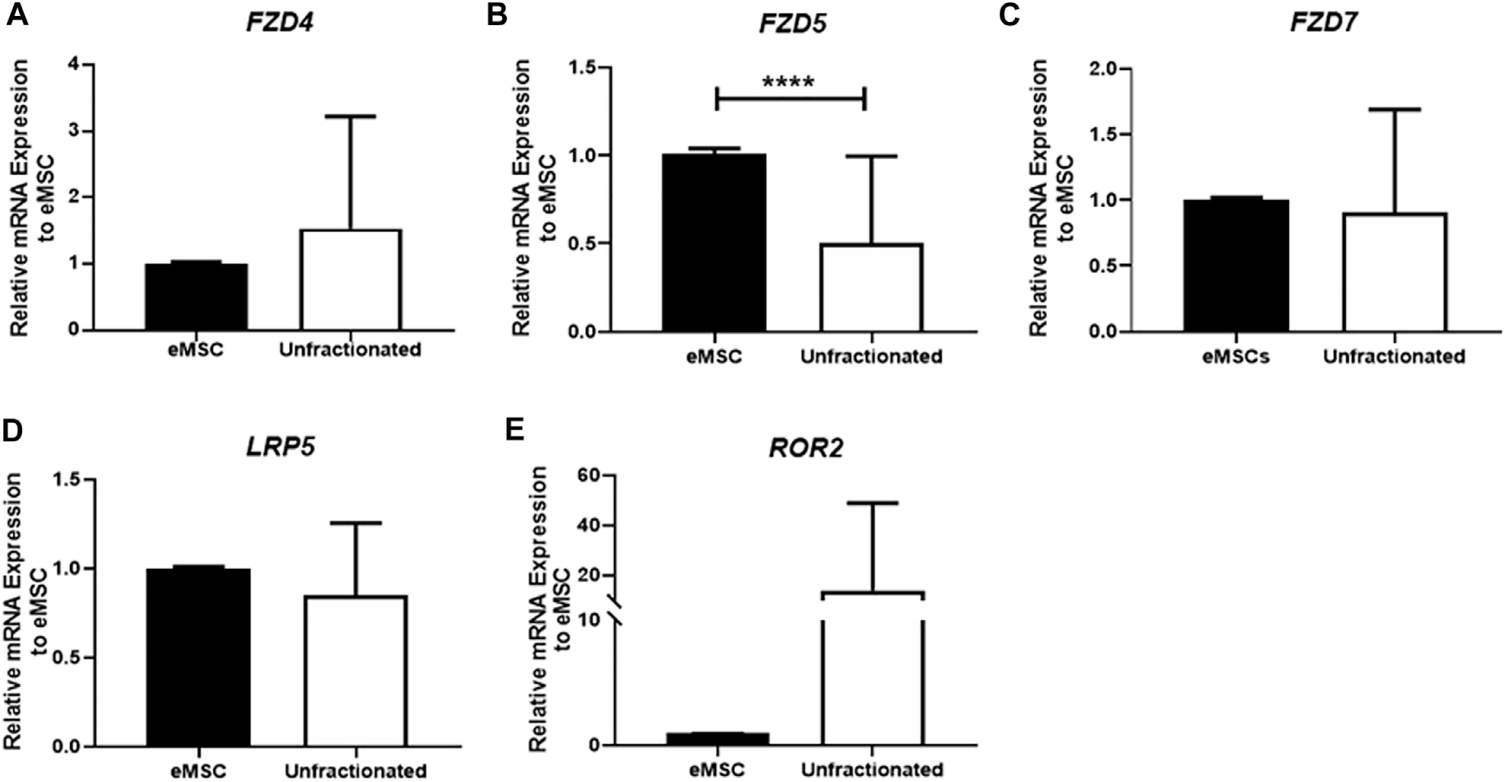
Gene expression of FZD receptors in unfractionated stromal cells, and eMSC. The relative gene expression of **(A)** *FZD4*, **(B)** *FZD5*, **(C)** *FZD7*, **(D)** *LRP5*, and **(E)** *ROR2* in eMSC (black bars) and unfractionated stromal cells (white bars) normalized to 18 S (n = 21). Results are presented as mean ± SD; ****p < 0.0001. Abbreviations: eMSC, endometrial mesenchymal stem cells; FZD, frizzled; LRP, low-density lipoprotein-related protein; ROR, receptor tyrosine kinase-like orphan receptors.

The gene expression of three *FZD* receptors (*FZD4*, *FZD5,* and *FZD7*) and WNT coreceptors (*LRP5, ROR2*) in unfractionated endometrial stromal cells and eMSC were assessed by qPCR. These receptors and coreceptors were selected based on previous reports that they could bind with WNT5A (Umbhauer et al., 2000; Zhou et al., 2017). The mRNA expression of *FZD4* (n = 21, Figure 1A)*, FZD7* (n = 21, Figure 1C), *LRP5* (n = 21, Figure 1D), and ROR2 (n = 21, Figure 1E) were similar between two populations. Only the gene expression of *FZD5* was significantly higher in the eMSC than the unfractionated stromal cells (eMSC: 1.01 ± 0.03 fold; unfractionated: 0.51 ± 0.49 fold, *p* < 0.0001, n = 21, Figure 1B).

**FIGURE 2:**
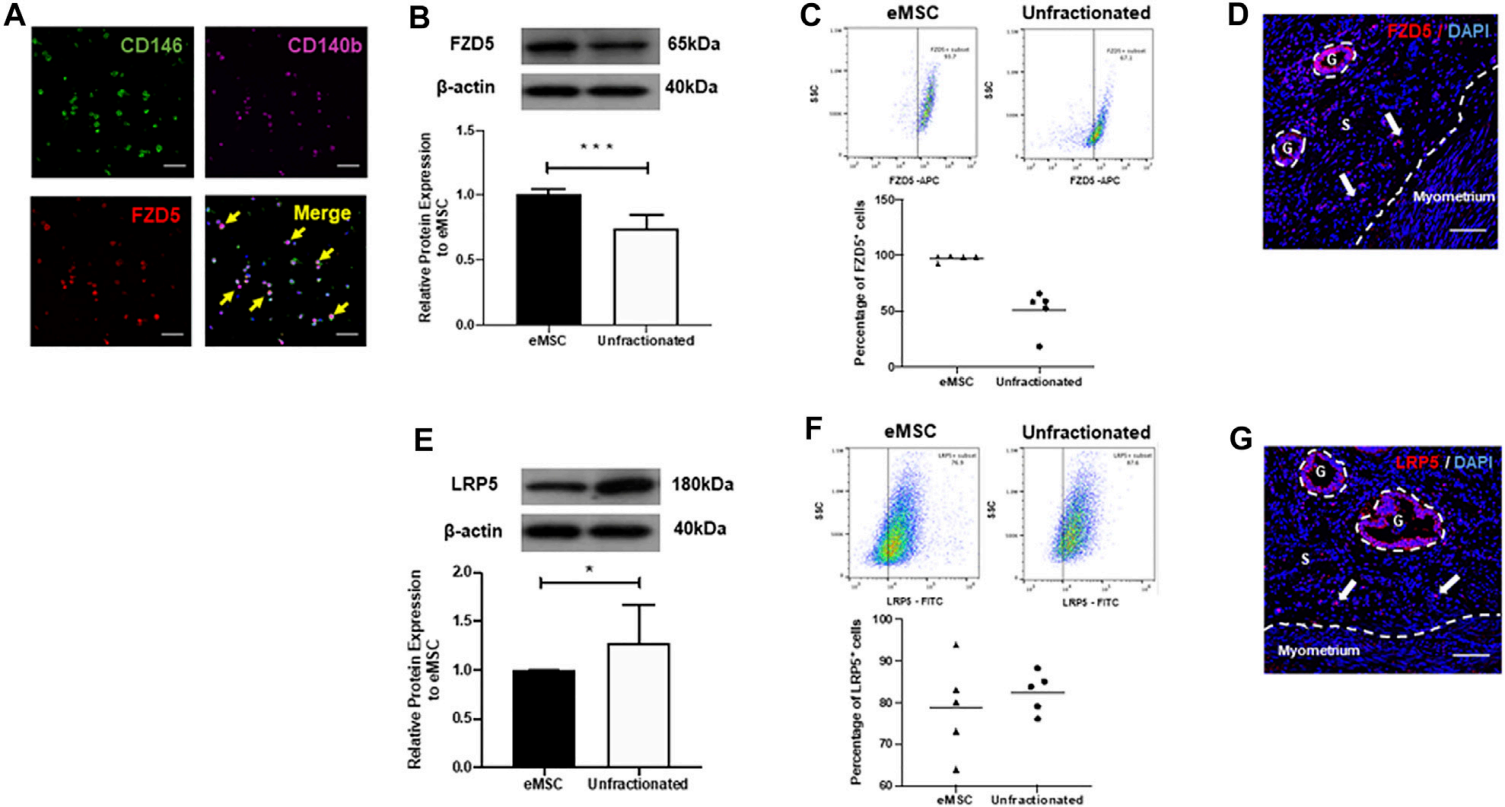
Expression of FZD5 and LRP5 in subpopulations of stromal cells and in human endometrial tissue. **(A)** The expression of CD146 (green), CD140b (pink), and FZD5 (red) in eMSC was confirmed by immunofluorescent staining (*yellow arrows*). Representative western blotting images and quantitative analysis of **(B)** FZD5 (n = 7) and **(E)** LRP5 (n = 6) protein expression in eMSC (black bars) and unfractionated stromal cells (white bars) normalized to β-actin. Percentage of **(C)** FZD5 or **(F)** LRP5 expressing cells using flow cytometry in eMSC (CD140b^+^CD146^+^ cells, triangles) and unfractionated stromal cells (dots, n = 5). **(D)** Human endometrial tissue stained with FZD5 (red). Stromal cells residing near the endometrial–myometrial junction express FZD5 (*white arrow*). **(G)** Human endometrial stained with LRP5 (red). Stromal cells residing near the endometrial–myometrial junction express LRP5 (*white arrow*). Results are presented as mean ± SD; *p < 0.05, ***p < 0.001. Scale bar: 100 µm. Abbreviations: eMSC, endometrial mesenchymal stem cells; FZD, frizzled; g, glands; LRP, low-density lipoprotein-related protein; s, stroma.

Colocalization of stem cell markers (CD140b and CD146) and the FZD5 receptor was confirmed by immunofluorescent staining (Figure 2A). Consistently, the protein expression of FZD5 was also significantly higher in the eMSC (1.01 ± 0.03 fold) when compared to the unfractionated stromal cells (0.75 ± 0.1 fold, *p* < 0.001, n = 7, Figure 2B). Quantitative analysis from flow cytometry revealed that 97.6 ± 1.2% of the eMSC expressed FZD5, while around 50.6 ± 8.4% of unfractionated stromal cells have FZD5 expression (n = 5, Figure 2C). In human endometrial tissue, FZD5 was expressed in glands and stroma (Figure 2D). In particular, stromal cells near the endometrial–myometrial junction expressed FZD5 (Figure 2D). For the coreceptor LRP5, unfractionated stromal cells (1.28 ± 0.16 fold) had higher protein expression when compared to eMSC (1.00 ± 0.01 fold, *p* < 0.05, n = 6, Figure 2E). Quantitative analysis from flow cytometry showed that around 78.7 ± 11.2% of eMSC and 82.5 ± 2.2% of unfractionated stromal cells have LRP5 expression (n = 5, Figure 2F). Glands and stromal cells in the human endometrium also expressed LRP5 (Figure 2G).

ROR2 has been documented to function as a WNT5A receptor mediating noncanonical WNT signaling (Mikels et al., 2009; Nishita et al., 2010). However, the protein expression of the coreceptor ROR2 in eMSC was undetectable by western blotting (data not shown) suggesting that ROR2 is unlikely to be involved in the regulation of eMSC.

### Neutralization of FZD5 Suppressed the Proliferation and Self-Renewal Activity of eMSC

**FIGURE 3:**
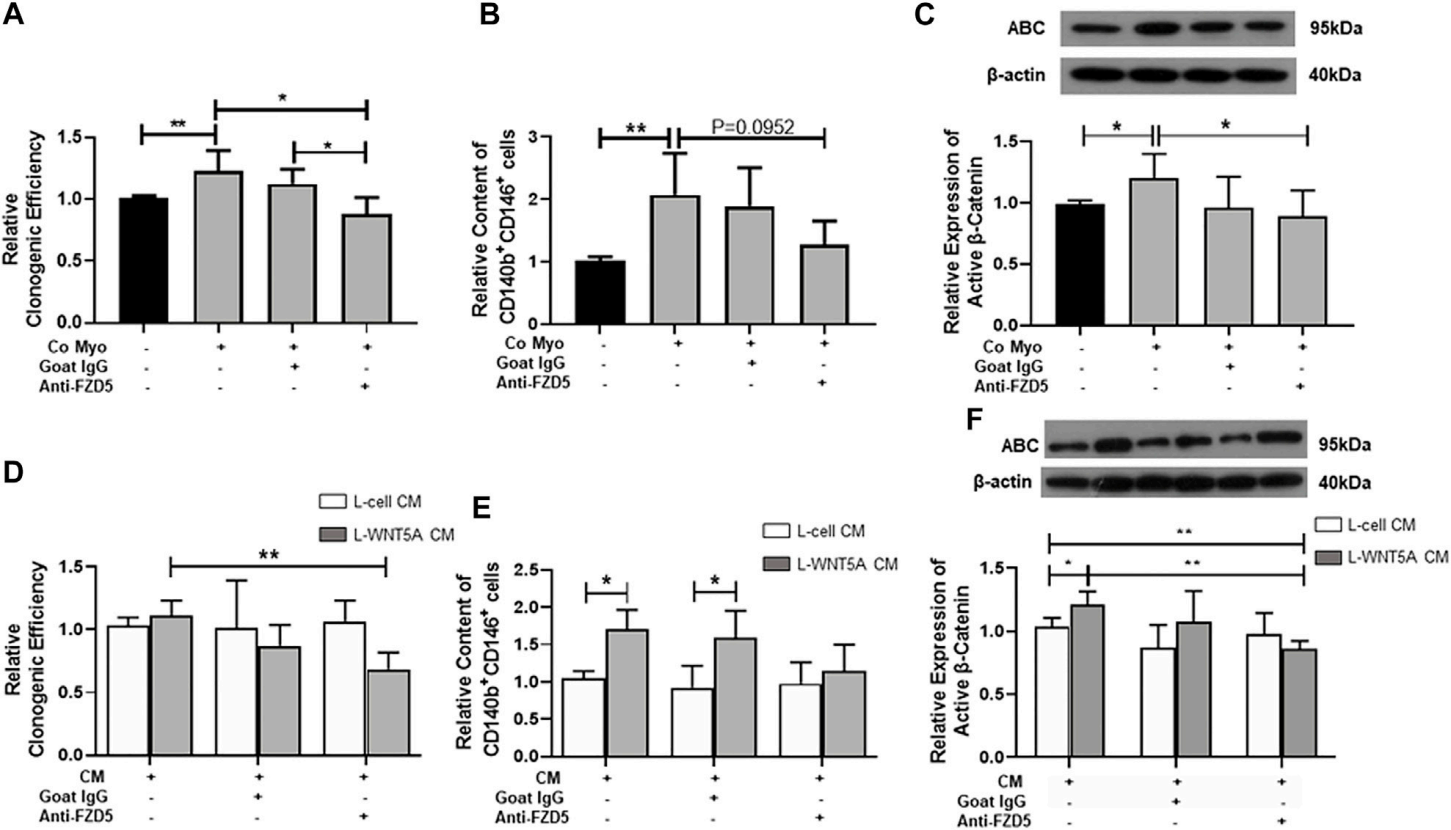
Effect of neutralizing FZD5 on eMSC activities. **(A)** Relative clonogenic efficiency of eMSC colonies in monoculture (black bars) and coculture with myometrial cells (gray bars) with goat IgG or 1 μg/ml neutralization FZD5 antibody (n = 5). **(B)** Relative proportion of CD140b^+^CD146^+^ cells after treatment (n = 5). **(C)** Relative expression of active β-catenin in eMSC in control (black bars) and co-culture with myometrial cells (gray bars) with goat IgG or 1 μg/ml neutralization FZD5 antibody (n = 7). **(D)** Relative clonogenicity of eMSC in L-cell CM (white bars) or L-Wnt5a CM (gray bars) with the addition of isotype goat IgG or 1 μg/ml neutralization FZD5 antibody (n = 5). **(E)** Relative clonogenicity of eMSC in L-cell CM (white bars) or L-Wnt5a CM (gray bars) with the addition of isotype goat IgG or 1 μg/ml neutralization FZD5 antibody (n = 5). **(F)** Relative expression of active β-catenin in eMSC in L-cell CM (white bars) or L-Wnt5a CM (gray bars) with the addition of isotype goat IgG or 1 μg/ml neutralization FZD5 antibody (n = 5, normalized to β-actin). Results are presented as mean ± SD, *p < 0.05, **p < 0.01. Abbreviations: CM, conditioned medium; co myo, co-culture with myometrial cells; eMSC, endometrial mesenchymal stem-like cells; FZD, frizzled.

To determine if FZD5 is a receptor for the activation of WNT/β-catenin signaling, we first cocultured myometrial cells with eMSC. As expected, the coculture significantly increased the relative cloning efficiency (1.23 ± 0.17 fold, *p* < 0.05, n = 5, Figure 3A), percentage of CD140b^+^CD146^+^ cells (2.07 ± 0.67 fold, *p* < 0.01, n = 5, Figure 3B), and relative expression of active β-catenin (1.2 ± 0.2 fold, *p* < 0.05, n = 7, Figure 3C) when compared to monoculture (cloning efficiency: 1.01 ± 0.02 fold; percentage of CD140b^+^CD146^+^ cells: 1.02 ± 0.05 fold; relative expression of active β-catenin: 0.99 ± 0.03 fold). Although the addition of neutralizing anti-FZD5 antibody did not reduce the phenotypic expression of eMSC (1.28 ± 0.38 fold, *p* = 0.09, Figure 3B), the treatment reduced the stimulatory effect of myometrial coculture on colony formation (0.88 ± 0.14 fold, *p* < 0.05, n = 5) and WNT/β-catenin signaling activation (0.89 ± 0.21 fold, *p* < 0.05, n = 7). No change was observed after the addition of goat IgG in the control group.

Next, L-Wnt5A CM was used to verify the ligand–receptor interaction in eMSC. L-cell CM was used as the control. Some components in L-cell can increase the clonogenic efficiency of eMSC when compared to the control (L-cell: 1.37 ± 0.3 fold, control: 1.05 ± 0.12 fold; *p* < 0.05, n = 5, Supplementary Figure S1B). The addition of anti-FZD5 antibody abolished the stimulatory effect of L-Wnt5A CM (anti-FZD5 antibody: 0.68 ± 0.13 fold; L-Wnt5A CM: 1.13 ± 0.1 fold, n = 5, *p* < 0.01, Figure 3D). No difference was observed in the percentage of CD140b^+^CD146^+^ cells between the control group (1.04 ± 0.09 fold, n = 5) and the L-cell CM-treated group (1.22 ± 0.36 fold, n = 5, Supplementary Figure S1C). L-Wnt5A CM-treated groups significantly increased the phenotype expression of eMSC when compared with those treated with or without goat IgG (L-Wnt5A CM: 1.71 ± 0.26 fold; L-cell CM with IgG: 0.92 ± 0.29 fold; L-Wnt5A CM with IgG: 1.6 ± 0.36 fold, n = 5, *p* < 0.05, Figure 3E). However, the expression of eMSC markers remained unchanged upon the addition of the anti-FZD5 antibody (1.15 ± 0.35 fold, n = 5, Figure 3E). The expression of active β-catenin elevated in the L-Wnt5A CM-treated group when compared to that in the L-cell group and declined with the supplement of anti-FZD5 antibody (L-cell CM: 1.03 ± 0.07 fold; L-Wnt5A CM: 1.21 ± 0.1 fold; L-Wnt5A with anti-FZD5 antibody: 0.86 ± 0.07 fold, n = 5, *p < 0.05, **p < 0.01, Figure 3F), demonstrating the changes of WNT/β-catenin signaling activities under different culture conditions.

### Gene Silencing of FZD5 Reduced the Active β-Catenin Activity in eMSC

**FIGURE 4:**
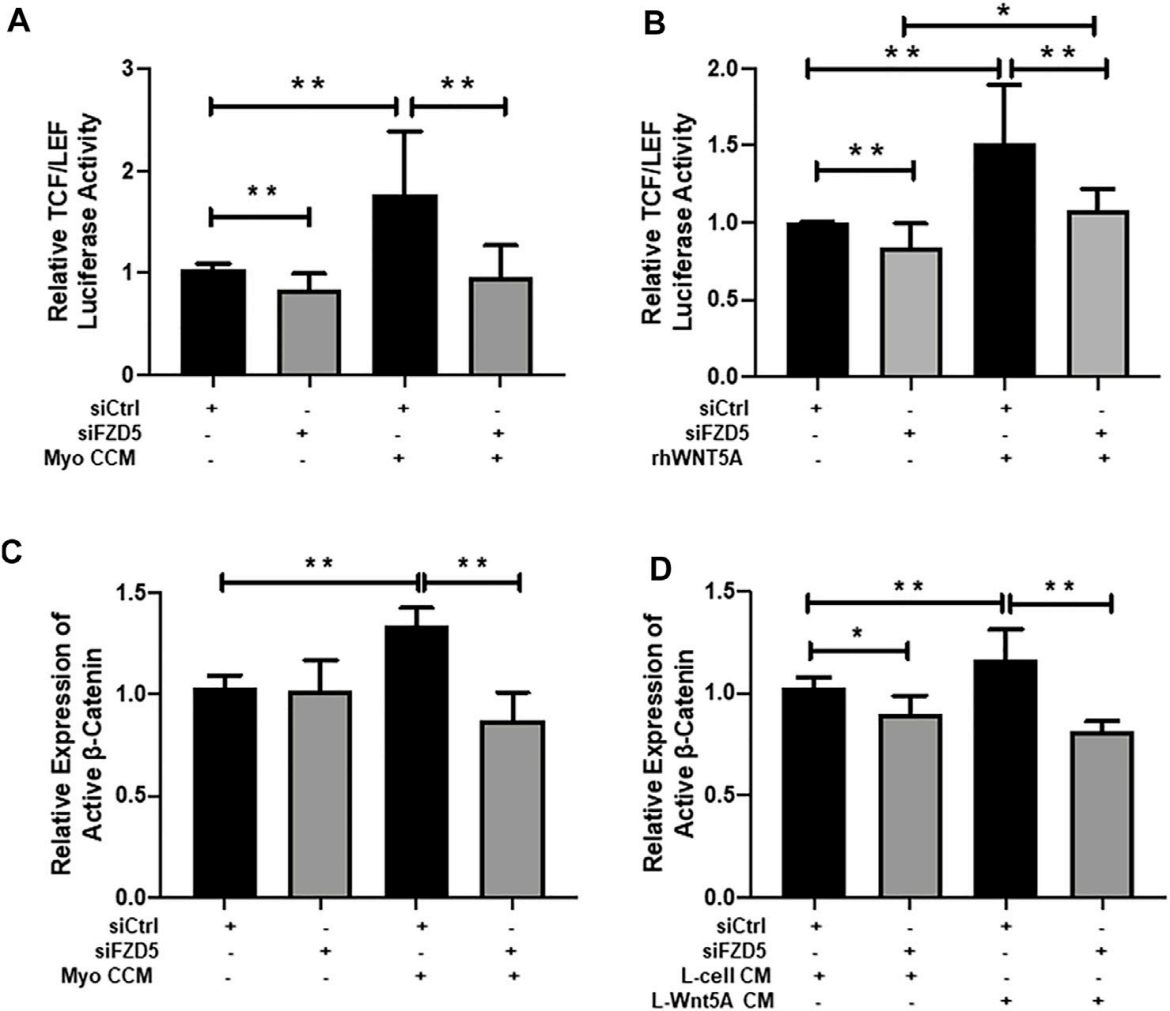
Effect of FZD5 silencing on WNT/β-catenin signaling. **(A)** The TCF/LEF luciferase activity of eMSC after transfection with scrambled control siRNA (siCtrl) (black bars), siRNA directed toward FZD5 (siFZD5) (gray bars) and cultured in myometrial CCM after transfection with siCtrl or siFZD5 (n = 6). **(B)** The TCF/LEF luciferase signal of eMSC after transfection with scrambled control siRNA (siCtrl) (black bars), siRNA directed toward FZD5 (siFZD5) (gray bars) and cultured with 0.01 μg/ml of rhWNT5A after transfection with siCtrl or siFZD5 (n = 6). **(C)** Relative expression of active β-catenin in eMSC after transfection with or without myometrial CCM (n = 5, normalized to β-actin). **(D)** Relative expression of active β-catenin in eMSC after transfection with or L-cell Wnt5a CM (n = 5). Results are presented as mean ± SD; *p < 0.05, **p < 0.01. Abbreviations: Myo CCM, myometrial concentrated conditioned medium; rhWNT5A, human recombinant WNT5A; eMSC, endometrial mesenchymal stem-like cells; FZD, frizzled.

To demonstrate that WNT5A-FZD5-β-catenin mediated the effects on eMSC, we evaluated the TCF/LEF luciferase activity after gene silencing of FZD5. Consistently, the addition of myometrial CCM or recombinant WNT5A protein significantly increased the relative luciferase activity of eMSC when compared to the corresponding control siRNA group (Myo CCM: 1.77 ± 0.62 fold, *p* < 0.01, n = 6, Figure 4A; rhWNT5A: 1.52 ± 0.38 fold, *p* < 0.01, n = 6, Figure 4B). FZD5-siRNA reduced the induction effect of TCF/LEF luciferase activity (siFZD5 + Myo CCM: 0.95 ± 0.31 fold, *p* < 0.01, Figure 4A; siFZD5 + rhWNT5A: 1.08 ± 0.14 fold, *p* < 0.01, Figure 4B).

The expression of active β-catenin in eMSC after transfection with FZD5-siRNA was also evaluated. Myometrial CCM (1.34 ± 0.09 fold) and L-Wnt5A CM (1.17 ± 0.15 fold) significantly increased the relative expression of active β-catenin when compared to the control siRNA (1.03 ± 0.06 fold, *p* < 0.01, n = 5, Figure 4C) or the L-cell siRNA group (1.03 ± 0.05 fold, *p* < 0.01, n = 5, Figure 4D). Similarly, knockdown of FZD5 decreased the stimulatory effect of myometrial CCM (0.87 ± 0.14 fold) and L-Wnt5A CM (0.81 ± 0.05 fold) on active β-catenin expression when compared to siFZD5 (1.02 ± 0.15 fold, *p* < 0.01, Figure 4C, Supplementary Figure S2) or the siFZD5 in the L-cell CM (0.9 ± 0.09 fold, *p* < 0.01, Figure 4D, Supplementary Figure S3) group.

### DKK-1 Reduced the WNT5A Stimulatory Effect in eMSC

**FIGURE 5:**
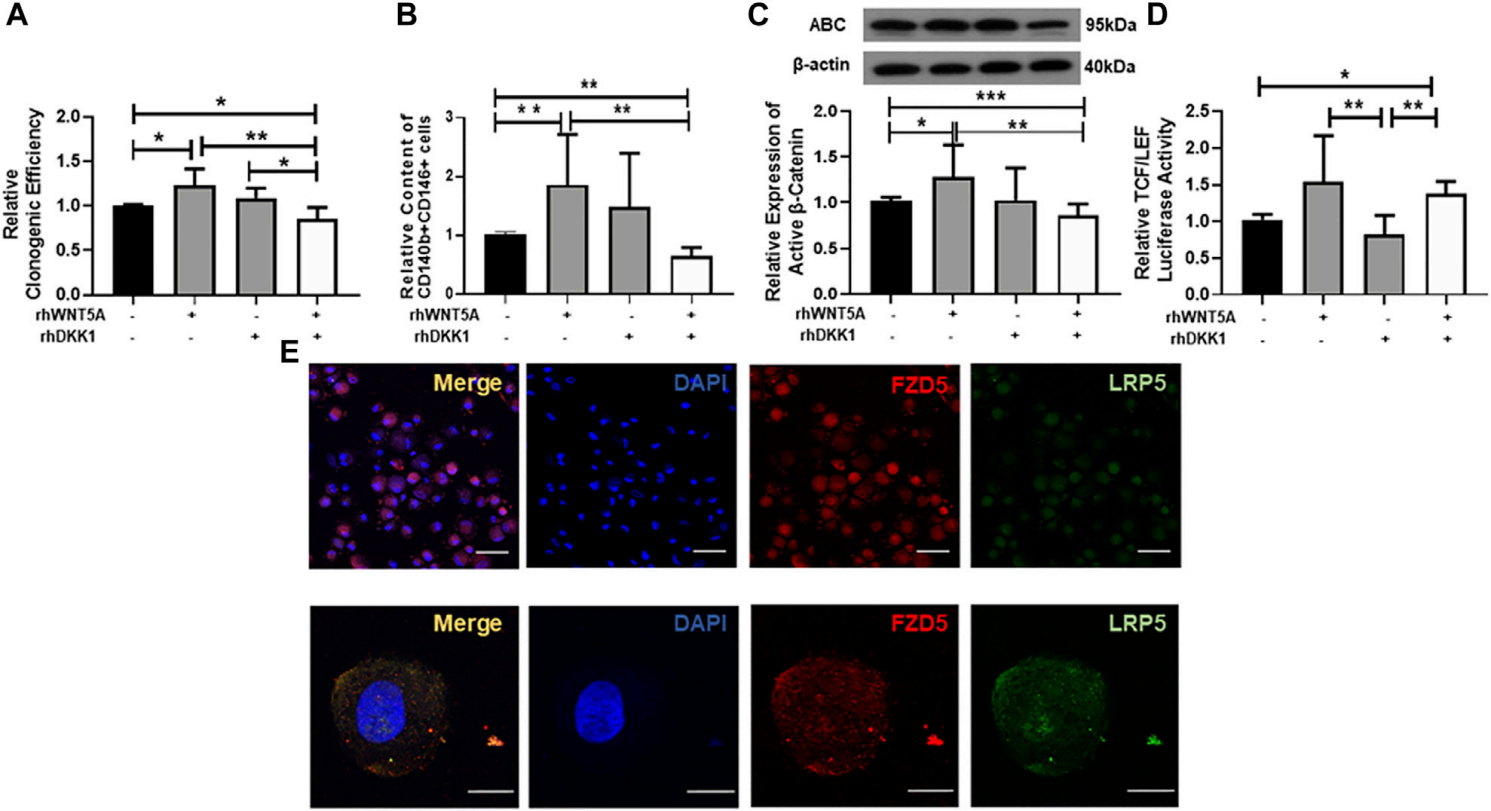
Effect of neutralizing LRP5 on eMSC activities. **(A)** Relative clonogenic efficiency of eMSC colonies in control (black bars), GM containing 0.01 μg/ml rhWNT5A or 0.1 μg/ml rhDKK1 protein (gray bars) and combination of 0.01 μg/ml rhWNT5A with 0.1 μg/ml rhDKK1 protein (n = 5). **(B)** Relative proportion of CD140b^+^CD146^+^ cells (n = 5). **(C)** Relative expression of active β-catenin in eMSC in control (black bars), GM containing 0.01 μg/ml rhWNT5A or 0.1 μg/ml rhDKK1 protein (gray bars) and combination of 0.01 μg/ml rhWNT5A with 0.1 μg/ml rhDKK1 protein (n = 7, normalized to β-actin). **(D)** The TCF/LEF luciferase signal of eMSC after treatment with growth medium containing 0.1 μg/ml rhDKK1 protein with or without 0.01 μg/ml rhWNT5A (gray bars) and 0.01 μg/ml rhWNT5A or rhDKK1 (white bars) (n = 7). **(E)** Coexpression of FZD5 (red) and LRP5 (green) on eMSC. Results are presented as mean ± SD, *p < 0.05, **p < 0.01. Scale bar: 50 µm (lower magnification) and 10 µm (higher magnification). Abbreviations: ABC, active β-catenin; DKK-1, Dickkopf-1; eMSC, endometrial mesenchymal stem-like cells; FZD, frizzled; GM, growth medium; LRP, low-density lipoprotein-related protein.

To investigate the function of the WNT coreceptor LRP5 on the maintenance of eMSC, we used the WNT antagonist Dickkopf-1 (DKK1) protein. Treatment with WNT5A protein increased the phenotypic expression of eMSC (1.86 ± 0.86 fold) compare to the control (1.02 ± 0.05 fold, *p* < 0.05, n = 6, Figure 5B). Addition of DKK1 and WNT5A protein significantly decreased the clonogenicity (0.86 ± 0.13 fold) and the proportion of eMSC (0.65 ± 0.15 fold) when compared to the WNT5A protein alone (clonogenicity: 1.23 ± 0.19 fold, *p* < 0.05, n = 6, Figure 5A; phenotypic expression: 1.45 ± 0.90, *p* < 0.01, Figure 5B). Similarly, the WNT5A-induced increase in active β-catenin expression was abolished upon the treatment of eMSC with DKK-1 (Wnt5A: 1.28 ± 0.36 fold; *p* < 0.05; Wnt5A + DKK1: 0.86 ± 0.12 fold; *p* < 0.01, n = 7, Figure 5C).

To investigate the role of LRP5 in regulating the WNT/β-catenin signaling, the TCF/LEF luciferase activities in eMSC were evaluated after treatment with DKK-1 protein. Treatment with DKK-1 and WNT5A proteins (1.38 ± 0.16 fold) or WNT5A alone (1.54 ± 0.63 fold) significantly increased the relative luciferase activity when compared to the DKK-1 protein group (0.82 ± 0.26 fold, *p* < 0.01, n = 7, Figure 5D). Dual immunofluorescent staining confirmed that the majority of the eMSC coexpress FZD5 and LRP5 (Figure 5E).

### WNT5A Mediates the Activation of eMSC by Interacting With FZD5 and LRP5

**FIGURE 6:**
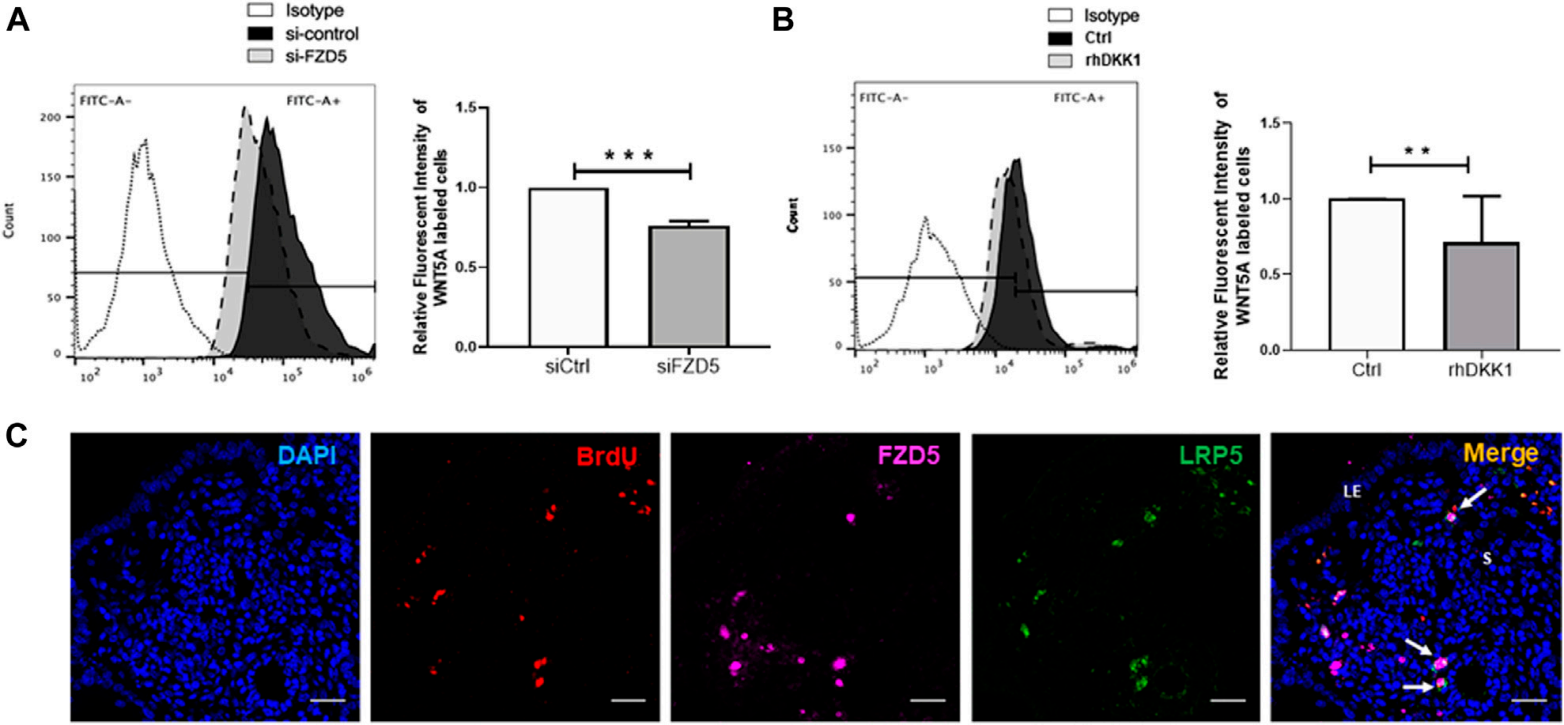
Bound signal of WNT5A with eMSC after FZD5 gene silencing and blocking of LRP5. **(A)** Representative images showing the peak shift of the fluorescent intensity in eMSC after transfection with control siRNA (siCtrl) (black) and siFZD5 (gray). Bar graph showing the relative fluorescent intensity of eMSC after transfection with siFZD5 (n = 7). **(B)** Representative images showing the peak shift of the fluorescent intensity in eMSC with (gray) or without (black) treatment with 0.1 μg/ml rhDKK1 protein. Bar graph showing the relative fluorescent intensity of eMSC after treatment with 0.1 μg/ml rhDKK1 (n = 7). **(C)** Representative immunofluorescence images showing the coexpression of FZD5 (pink) and LRP5 (green) in label retaining stromal cells (LRSCs: BrdU^+^ cells, red) at 6 weeks chase (white arrow). Scale bar: 20 µm. Results are presented as mean ± SD, **p < 0.01, ***p < 0.001. Abbreviations: BrdU, bromodeoxyuridine; DKK-1, Dickkopf-1; eMSC, endometrial mesenchymal stem-like cells; FZD, frizzled; LE, luminal epithelium; s, stroma.

To confirm the WNT5A–FZD5 interaction, we used fluorescence-labeled WNT5A protein to assess the bound signal on eMSC after siFZD5 transfection. The fluorescent intensity bound WNT5A on eMSC reduced after transfection with siFZD5 (siCtrl: 1.03 ± 0.09 fold; siFZD5: 0.75 ± 0.03 fold, *p* < 0.001, n = 7, Figure 6A), suggesting FZD5-expressed eMSC can interact with WNT5A.

Next, DKK-1 was used to assess the response of LRP5 on eMSC. The relative fluorescent intensity of WNT5A-labeled eMSC reduced after the addition of DKK-1 protein (control: 1.01 ± 0.02 fold; rhDKK1: 0.71 ± 0.31 fold, *p* < 0.01, n = 7, Figure 6B).

### Label-Retaining Stromal Cells in Mouse Endometrium Express FZD5 and LRP5

**FIGURE 7:**
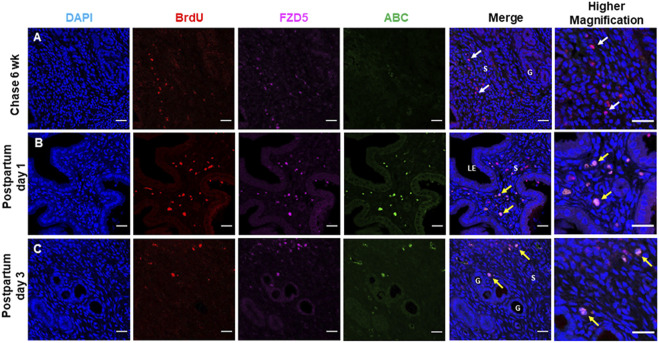
Label retaining stromal cells express FZD5 receptor and active β-catenin. Representative immunofluorescence images showing the label retaining stromal cells (LRSCs: BrdU^+^ cells, red) at **(A)** 6 weeks chase (*white arrow*), **(B)** PPD1, and **(C)** PPD3 colocalized with FZD5 (pink). Triple staining of BrdU/ABC/FZD5 (*yellow arrow*) in PPD1 and PPD3. Scale bar: 20 μm, 10 µm. Abbreviations: ABC, active β-catenin; BrdU, bromodeoxyuridine; FZD, frizzled; G, gland; LE, luminal epithelium; PPD, postpartum day; s, stroma.

Using the BrdU label, the LRC approach has identified stem/progenitor cells in the mouse endometrium (Chan and Gargett, 2006). Label-retaining stromal cells (LRSCs) were present after 6 weeks chase, and the percentage of these cells remained steady until parturition (Cao et al., 2015). In the mouse endometrial tissue, LRSCs (BrdU^+^ cells) were found to coexpress with FZD5 and LRP5 at 6 weeks chase (Figure 6C). Activated LRSCs in the postpartum endometrium expressed Wnt/β-catenin signal pathway components during uterine remodeling (Cao et al., 2015). The active β-catenin expression on LRSCs was only detected in the postpartum endometrium (Figure 7), and these ABC^+^ cells coexpressed FZD5 and BrdU, indicating that endometrial stem-like cells require the activation of Wnt/β signaling for tissue regeneration.

## Discussion

Stem cells remain quiescent and undifferentiated when situated within the niche. Most of the work in the field of endometrium stem/progenitor cells focuses on identifying, characterizing, and locating the origin of the cells (Gargett et al., 2016; Santamaria et al., 2018). The molecular mechanisms controlling stem cell renewal and differentiation remain unknown. To gain a better understanding of the interplay between endometrial stem/progenitor cells and their niche, we have identified myometrial cells as a niche component of eMSC. Coculture of myometrial cells or myometrial CCM with eMSC enhanced proliferation and renewal activities when compared to monoculture or cultured with growth medium alone. In addition, the participation of WNT/β-catenin signaling as a molecular component in the eMSC niche was observed in mice and humans (Cao et al., 2015, 2019; Xu et al., 2020). The impact of WNT/β-catenin signaling in stem cell regulation is well documented (Grigoryan et al., 2008; Haegebarth and Clevers, 2009; Bukowska et al., 2015). In the human endometrium, we have demonstrated that myometrial-secreted WNT5A can activate the WNT/β-catenin pathway in eMSC (Cao et al., 2019).

WNT5A has been proven to be a classic noncanonical WNT ligand (Yamanaka et al., 2002; Dejmek et al., 2006). However, WNT5A can activate the canonical WNT pathway depending on the receptor context (Mikels and Nusse, 2006). For example, frizzled receptors such as FZD4, FZD5, and FZD7 can interact with WNT5A and activate canonical and noncanonical pathways (Umbhauer et al., 2000; Mikels and Nusse, 2006; Hii et al., 2015). Overall, endometrial stromal cells express Frizzled genes, and the expression was similar among the stromal subpopulations. Interestingly, eMSC contain a higher expression of *FZD5* than unfractionated stromal cells. Protein evaluations further confirmed that this receptor was abundantly expressed by eMSC. Knockdown of *FZD5* expression and blocking LRP5 significantly reduced the stimulatory effect of WNT5A on eMSC, providing evidence that these receptors can respond to WNT ligands in the endometrial microenvironment. Although LRP5 protein is higher in unfractionated stromal cells than eMSC, FZD5 and LRP5 have coexpression in a significant proportion of the eMSC population, suggesting the possibility of FZD5–LRP5 interaction in eMSC maintenance.

FZD5 is a common noncanonical WNT receptor (Ishikawa et al., 2001; Arderiu et al., 2014; Kim et al., 2019), and the blocking of WNT5A–FZD5 signaling pathway can inhibit the PKC pathway (Weeraratna et al., 2002). A recent study has demonstrated FZD5 as a regulator in the maintenance of human mesenchymal stem/stromal cells (hMSCs) (Harada et al., 2021). The β-catenin-independent WNT5A-FZD5 signaling preserved the properties of hMSCs by preventing cell senescence. Nevertheless, FZD5 can also activate the WNT/β-catenin signaling pathway in hMSCs (Kolben et al., 2012). It has been reported that WNT5A can act *via* FZD5 to initiate an intracellular pathway leading to the accumulation of β-catenin (He et al., 1997). The FZD–LRP complex can regulate the activation of LRP5/β-catenin signaling (Hua et al., 2018; Chen et al., 2019). This FZD–LRP interaction also blocks the activation of noncanonical WNT pathways by inhibiting FZD-mediated signaling (Ren et al., 2015). Therefore, we postulate that the FZD5 and LRP5 expressing eMSC can activate the WNT/β-catenin pathway by interacting with WNT ligands produced by niche cells, in particular the response to WNT5A.

Myometrial coculture enhanced the clonogenic activity and phenotypic expression of stem cell markers (CD140b and CD146) in eMSC. Similar stimulatory effect was observed when L-Wnt5A CM was used. The addition of anti-FZD5 antibody reduced the stimulatory action of myometrial coculture on colony formation and self-renewal of eMSC. When L-Wnt5a CM was blocked with anti-FZD5, a decreasing trend in the percentage of eMSC was also observed, but the result did not reach significance due to high variation between individuals. The use of antibodies may not efficiently neutralize binding of the ligand to the receptor. Thus, knockdown of the receptor with gene silencing will be more specific. Indeed, the stimulatory effect of myometrial CCM or recombinant WNT5A significantly reduced the TCF/LEF transcriptional activities of eMSC upon the knockdown with FZD5-siRNA. From the other aspect, blocking the LRP5 coreceptor also significantly reduced the clonogenicity and proportion of eMSC due to the inhibition of the canonical WNT signaling pathway. Although DDK1 can inhibit the effect of LRP5 and LRP6, we only evaluated LRP5 in this study because it was reported that WNT5A may activate the Wnt/β-catenin pathway in the presence of the LRP5 coreceptor and FZD5 (He et al., 1997). The role of LRP6 in eMSC regulation remains to be explored.

Coexpression of FZD5 and LRP5 was also observed in mouse endometrial stem/progenitor cells. According to our previous findings, BrdU^+^ stromal cells after 6 weeks chase were termed LRSCs (Cao et al., 2015). The LRC approach can trace quiescent cells in the mouse endometrium, and hence we can study the changes of these functional LRSCs during pregnancy and postpartum. In addition, these FZD5^+^ LRSCs expressed active β-catenin at postpartum days 1 and 3, a period of dynamic physiological regeneration, indicating that WNT/β-catenin signaling is involved in stem cell activation during endometrial remodeling. WNT5A is an important Wnt ligand in female reproduction and is expressed in both the epithelial and stromal cells of uterine tissue (Hou et al., 2004; Latifi et al., 2018). These activities seen in WNT5A-activated cells fit well with our observation that endometrial LRSCs express FZD5, a prospective receptor for WNT5A. Whether WNT5A directly interacts with FZD5 expressing LRSC for uterine regeneration is yet to be determined. We postulate that FZD5 may also be a key molecule in regulating the stem cell fate in the mouse endometrium.

Overall, our study demonstrated that the interaction of WNT5A, FZD5, and LRP5 can regulate the proliferation and self-renewal of eMSC through the activation of the WNT/β-catenin signaling pathway. Further studies on the regulatory molecules can be done to develop an efficient protocol for the culture expansion of eMSC for cell-based therapy.

## Data Availability

The original contributions presented in the study are included in the article/Supplementary Material, further inquiries can be directed to the corresponding authors.
