# Supplementary figures and images for: WNT5A Interacts With FZD5 and LRP5 to Regulate Proliferation and Self-Renewal of Endometrial Mesenchymal Stem-Like Cells
Source: Front Cell Dev Biol. 2022 Feb 17;10:837827. doi: 10.3389/fcell.2022.837827 (PMC8919396; doi:10.3389/fcell.2022.837827)

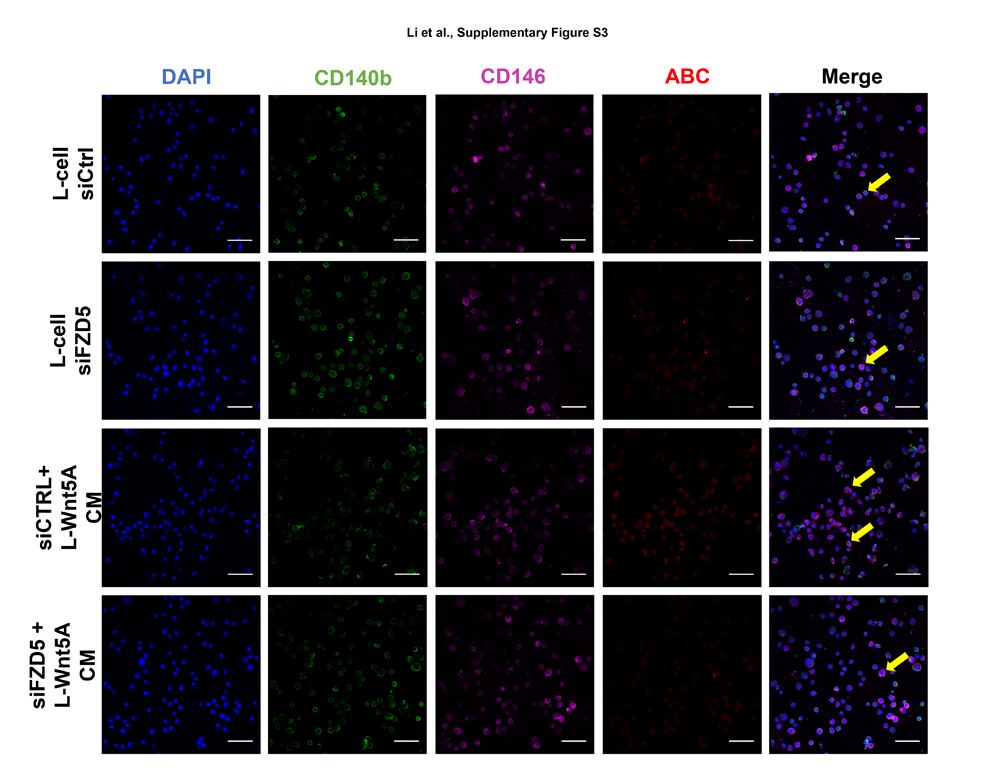

Supplement: Supplementary file 1 [file Figure10.TIF]

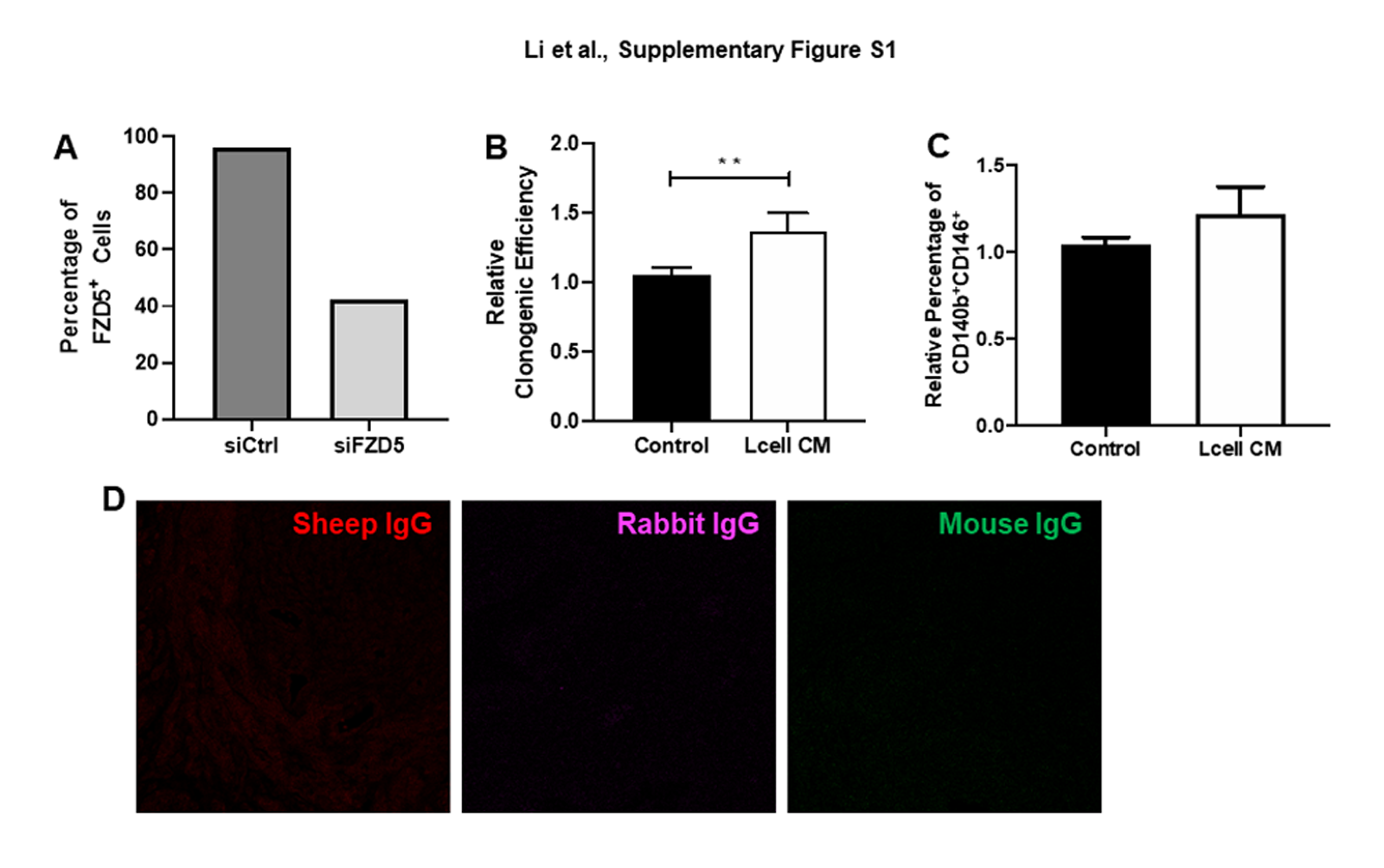

Supplement: Supplementary file 2 [file Figure8.TIF]

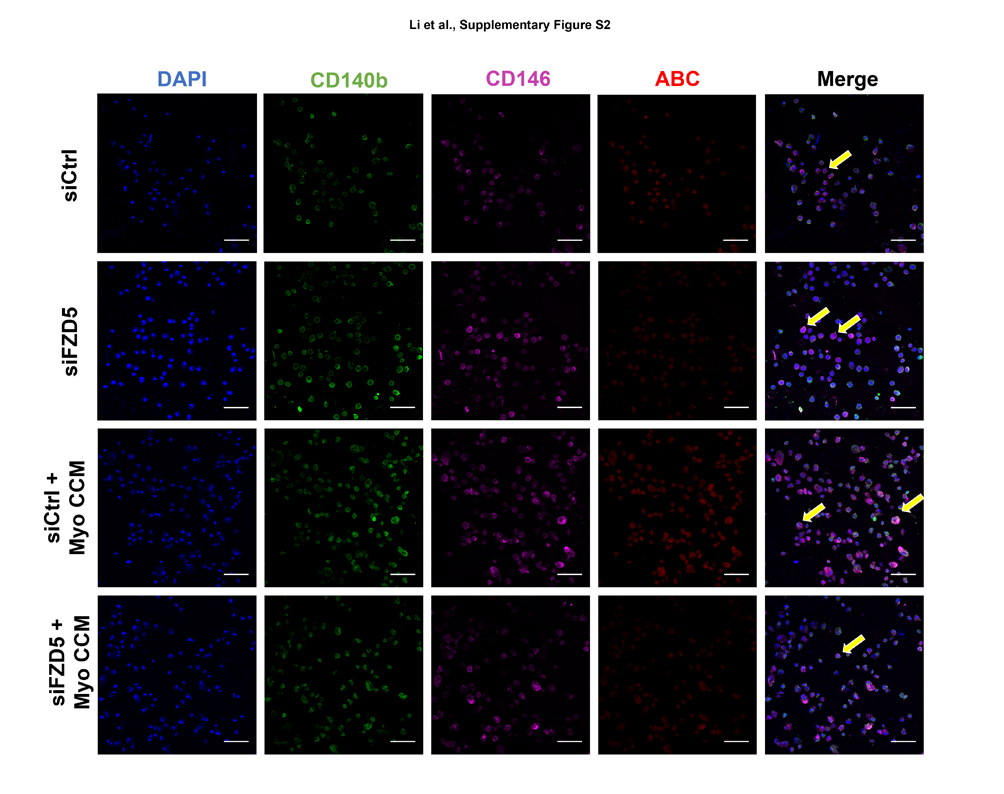

Supplement: Supplementary file 4 [file Figure9.TIF]
