# Supplementary material for: WNT5A Interacts With FZD5 and LRP5 to Regulate Proliferation and Self-Renewal of Endometrial Mesenchymal Stem-Like Cells
Source: Front Cell Dev Biol. 2022 Feb 17;10:837827. doi: 10.3389/fcell.2022.837827 (PMC8919396; doi:10.3389/fcell.2022.837827)
Supplement: Supplementary file 3 [file DataSheet2.docx]

**Li et al. Supplementary Methods**

**Immunofluorescent Staining**

For immunofluorescent staining of cells, ~8000 cells eMSCs after culture for 15 days under different treatments were trypsinized and use for cytospin. The cells were centrifuged at 12000 rpm for 10 minutes and fixed with 4% paraformaldehyde for 10 minutes. While for paraffin sections (5 µm) from human or mouse endometrial tissues were dewaxed with xylene, rehydrated with descending alcohol series and then in water before undergoing antigen retrieval using an antigen retrieval buffer (Dako) in a microwave oven. For BrdU staining, the slides were denatured with 0.1N HCL for 45 minutes. Permeabilization was performed using 0.1% Triton X-100 for 10 minutes and non-specific binding was blocked with 10% serum of the host species of the secondary antibody for 1 hour. Primary antibodies (Supporting Information Table S2) or isotype-matched control antibodies were incubated overnight at 4°C followed by the corresponding secondary antibodies (Supporting Information Table S2) for one hour. The cell nuclei were stained with DAPI (Invitrogen) and mounted with fluorescent mounting medium (Dako, Glostrup, Denmark). The slides were washed with PBST between steps and all steps were conducted at room temperature unless specified. Multi-spectrum fluorescence images were acquired using a LSM 700 inverted confocal microscope and a LSM ZEN 2010 software (Carl Zeiss, Munich, Germany) at the Centre for PanorOmic Sciences (CPOS) Imaging and Flow Cytometry Core, The University of Hong Kong. For quantification analysis, the total cell number and number of triple-positive cells were counted. At least 500 cells were counted from each sample.

**RNA extraction, reverse transcription and quantitative polymerase chain reaction (qPCR)**

Quantitative real-time polymerase chain reaction (qPCR) with Taqman probes was used (Addition File: Table S4). Total RNA of differentiated and control cells were extracted using the Absolutely RNA microprep kit (Stratagene, Agilent Technologies, La Jolla, USA). RNA concentrations were determined by a NanoDrop (NanoDrop 2000/2000c, ThermoFisher Scientific). Reverse transcription of RNA to cDNA was performed using the High Capacity cDNA Reverse Transcription Kit (Applied Biosystem, Foster City, USA). Real-time PCR was performed with a 7500 Real-Time PCR system (Applied Biosystems) using the following thermal cycle conditions: 50℃ for 2 minutes, then 95℃ for 10 minutes, followed by 40 cycles of 95℃ 15 seconds and 60℃ for 1 minute. All the experiments were performed in triplicates. The results are presented as relative expression compared with internal control 18S and calculated based on the comparative 2^-ΔΔct^ method.

**Western Blotting**

Proteins were extracted using lysis buffer (Invitrogen) with proteinase inhibitor (Merck, Darmstadt, Germany) and phosphatase inhibitor (Merck). The protein samples (30-70 µg) were mixed with 5X SDS loading dye (60 mM Tris-HCl pH 6.8, 2% SDS, 0.1% bromophenol blue, 25% glycerol and 5% β-mercaptoethanol) and denatured at 95°C for 10 minutes. The samples were then subjected to sodium dodecyl sulfate polyacrylamide gel electrophoresis and transferred to polyvinylidene difluoride (PVDF) membranes. The membranes were blocked with 5% skim milk (Nestle, Vevey, Switzerland) in PBS containing 0.1% Tween-20 (PBST) for 30 minutes and incubated with primary antibodies at appropriate concentrations (Table S3) overnight at 4°C. The membranes were stained with appropriate horseradish peroxidase-conjugated secondary antibodies (Table S3) for one hour. The protein bands were visualized by enhanced luminal-based chemiluminescence (Westsave UP™; AbFrontier, Seoul, Korea). The expression of the target proteins was calculated relative to the housekeeping protein β-actin. The scanned Western blot bands were quantified densitometrically and the values were normalized to the amount of β actin using the Image J software (US National Institutes of Health, USA).

**Multi-colour flow cytometry**

To assess the expression of LRP5 on different subpopulations of endometrial stromal cells or eMSCs, cells were incubated with APC-conjugated anti-CD146 (5 µg/ml, 541-10B2 clone, Mouse IgG1, Miltenyi Biotech), PE-conjugated anti-PDGFRβ (CD140b, 2.5 µg/ml, PR7212 clone, Mouse IgG1, R&D Systems) and anti-LRP5 (1 mg/ml, Goat IgG, Abcam) antibodies for 45 min at 4°C. Cell were washed with 0.1% BSA/PBS and incubated with secondary antibody goat anti-rabbit 488 (5 µg/ml, Invitrogen) for 45 min at 4°C.

To assess the expression of FZD5, cells were incubated FITC-conjugated anti-CD146 (5 µg/ml, OJ79C clone, mouse IgG1, ThermoFisher Scientific) and PE-conjugated anti-PDGFRβ (CD140b, R&D Systems) antibodies for 45 min on ice. The cells were then washed, fixed with 4% paraformaldehyde for 15 minutes and permeabilized for 15 minutes. Next, the cells were labeled with rabbit polyclonal anti-FZD5 antibody (5 µg/ml, Abcam, Cambridge, UK) in the dark for 45 minutes at 4°C. After washing, the cells were stained with goat anti-rabbit secondary antibody conjugated to allophycocyanin (1 µg/ml, ThermoFisher Scientific) in the dark for 45 minutes at 4°C.

Isotype matched controls were included for each antibody. Following the final washing step, the labeled cells were analyzed by a CytoFlex™ flow cytometer (Beckman Coulter, CA, USA). Data were analyzed with the FlowJo software (Tree Star Inc., OR, USA).
