# Supplementary material for: WNT5A Interacts With FZD5 and LRP5 to Regulate Proliferation and Self-Renewal of Endometrial Mesenchymal Stem-Like Cells
Source: Front Cell Dev Biol. 2022 Feb 17;10:837827. doi: 10.3389/fcell.2022.837827 (PMC8919396; doi:10.3389/fcell.2022.837827)
Supplement: Supplementary file 5 [file DataSheet1.docx]

**Supplementary Table S1** - Age of women, menstrual phase, and reason of hysterectomy in those who donated full thickness endometrial samples.

|  | Age | Menstrual Phase | Pathology |
| --- | --- | --- | --- |
| 1 | 53 | Proliferative | Leiomyomas |
| 2 | 48 | Proliferative | Leiomyomas |
| 3 | 45 | Proliferative | Leiomyomas |
| 4 | 46 | Proliferative | Leiomyomas |
| 5 | 43 | Proliferative | Adenomyosis |
| 6 | 46 | Proliferative | Leiomyomas |
| 7 | 48 | Proliferative | Adenomyosis |
| 8 | 47 | Proliferative | Leiomyomas |
| 9 | 47 | Proliferative | Leiomyomas |
| 10 | 49 | Proliferative | Leiomyomas |
| 11 | 51 | Proliferative | Leiomyomas |
| 12 | 47 | Proliferative | Leiomyomas |
| 13 | 47 | Proliferative | Leiomyomas |
| 14 | 49 | Proliferative | Leiomyomas |
| 15 | 48 | Proliferative | Leiomyomas |
| 16 | 44 | Proliferative | Adenomyosis + leiomyomas |
| 17 | 41 | Proliferative | Leiomyomas |
| 18 | 49 | Proliferative | Leiomyomas |
| 19 | 50 | Proliferative | Leiomyomas |
| 20 | 47 | Proliferative | Leiomyomas |
| 21 | 47 | Proliferative | Adenomyosis |
| 22 | 47 | Proliferative | Leiomyomas |
| 23 | 45 | Proliferative | Leiomyomas |
| 24 | 49 | Secretory | Leiomyomas |
| 25 | 49 | Secretory | Adenomyosis |
| 26 | 52 | Secretory | Leiomyomas |
| 27 | 49 | Secretory | adenomyosis |
| 28 | 46 | Secretory | Leiomyomas |
| 29 | 49 | Secretory | Leiomyomas |
| 30 | 44 | Secretory | Leiomyomas |
| 31 | 41 | Secretory | Leiomyomas |
| 32 | 46 | Secretory | Adenomyosis |
| 33 | 45 | Secretory | Leiomyomas |
| 34 | 46 | Secretory | Leiomyomas |
| 35 | 47 | Secretory | Leiomyomas |
| 36 | 47 | Secretory | Leiomyomas |
| 37 | 48 | Secretory | Leiomyomas |
| 38 | 44 | Secretory | Leiomyomas |
| 39 | 44 | Secretory | Leiomyomas |
| 40 | 43 | Secretory | Adenomyosis + leiomyomas |
| 41 | 41 | Secretory | Leiomyomas |

**Supplementary Table S2 - List of primary and secondary antibodies used for immunofluorescent staining**

| **Primary Antibodies (IF)** | **Dilution** | **Source** |
| --- | --- | --- |
| **BrdU:** sheep polyclonal to BrdU | 1:800 | Abcam |
| **CD146:** mouse monoclonal to CD146 | 1:100 | Novus |
| **Frizzled 5:** rabbit polyclonal to frizzled 5 | 1:500 | Abcam |
| **LRP5:** goat polyclonal to LRP5 | 1:100 | Abcam |
| **PDGFRb:** goat polyclonal to PDGFRb | 1:200 | R&D Systems |
| **Secondary Antibodies (IF)** | **Dilution** | **Source** |
| Donkey anti-goat Alexa Fluor 555 | 1:200 | Invitrogen |
| Donkey anti-rabbit Alexa Fluor 568 | 1:200 | Invitrogen |
| Donkey anti-rabbit Alexa Fluor 647 | 1:200 | Invitrogen |
| Donkey anti-sheep Alexa Fluor 555 | 1:200 | Invitrogen |
| Rabbit anti-goat Alexa Fluor 488 | 1:200 | Invitrogen |
| Rabbit anti-mouse Alexa Fluor 488 | 1:200 | Invitrogen |

**Supplementary Table S3- List of primary antibodies used for western blotting**

| **Primary Antibodies (WB)** | **Isotype** | **Dilution** | **Source** |
| --- | --- | --- | --- |
| **FZD5:**  rabbit polyclonal to frizzled 5. | Rabbit IgG1 | 1:500 | Abcam |
| **LRP5:** goat polyclonal LRP5. | Goat IgG | 1:500 | Abcam |
| **β-actin**: mouse monoclonal beta actin; clone AC-15. | Mouse IgG1 | 1:10000 | Sigma-Aldrich |

| **Secondary Antibodies (WB)** | **Dilution** | **Source** |
| --- | --- | --- |
| Mouse horseradish peroxidase | 1:5000 | GE Healthcare |
| Goat horseradish peroxidase | 1:5000 | GE Healthcare |
| Rabbit horseradish peroxidase | 1:5000 | GE Healthcare |

**Supplementary Table S4 – Taqman probes used for qPCR**

| **Gene Name *(Gene Symbol)*** | **Taqman Probes** |
| --- | --- |
| Frizzle 4 *(FZD4)* | Hs00201853_m1 |
| Frizzled 5 *(FZD5)* | Hs00258278_s1 |
| Frizzled 7 *(FZD7)* | Hs00275833_s1 |
| Low-density lipoprotein receptor-related protein 5 *(LRP5)* | Hs00182031_m1 |
| Receptor tyrosine kinase-like orphan receptor 2 *(ROR2)* | Hs00896176_m1 |
